# Incoherent Nuclear Resonant Scattering from a Standing Spin Wave

**DOI:** 10.1038/s41598-018-29596-z

**Published:** 2018-07-26

**Authors:** Jakob Gollwitzer, Lars Bocklage, Kai Schlage, Marcus Herlitschke, Hans Christian Wille, Olaf Leupold, Christian F. Adolff, Guido Meier, Ralf Röhlsberger

**Affiliations:** 1 Institute for Applied Physics, Universität Hamburg, Jungiusstrasse 11, 20355 Hamburg, Germany; 2 Deutsches Elektronen-Synchrotron DESY, Notkestrasse 85, 22607 Hamburg, Germany; 3 The Hamburg Centre for Ultrafast Imaging, Luruper Chaussee 149, 22761 Hamburg, Germany; 4 Max-Planck Institute for the Structure and Dynamics of Matter, Luruper Chaussee 149, 22761 Hamburg, Germany

## Abstract

We introduce a method to study the spatial profiles of standing spin waves in ferromagnetic microstructures. The method relies on Nuclear Resonant Scattering of ^57^Fe using a microfocused beam of synchrotron radiation, the transverse coherence length of which is smaller than the length scale of lateral variations in the magnetization dynamics. Using this experimental method, the nuclear resonant scattering signal due to a confined spin wave is determined on the basis of an incoherent superposition model. From the fits of the Nuclear Resonant Scattering time spectra, the precessional amplitude profile across the stripe predicted by an analytical model is reconstructed. Our results pave the way for studying non-homogeneous dynamic spin configurations in microstructured magnetic systems using nuclear resonant scattering of synchrotron light.

## Introduction

Understanding the spatial profile of a dynamic spin configuration in laterally confined magnetic systems is of fundamental importance for their potential use in functional devices. Permalloy (Ni_80_Fe_20_) thin films structured by lithographic methods have become a testbed for exploring new concepts in the emerging field of magnonics that deals with the use of spin waves for magnetic data processing^1^. Among many other applications, permalloy micro- and nanostructures have been used to build magnonic logic gates^2^, spin wave guides^3^, and spin multiplexers^4^. The spatial profile of the dynamic spin configuration in such systems is most commonly mapped with inelastic scattering methods such as Brillouin Light Scattering^5^ and X-Ray Magnetic Circular Dichroism^6^. These methods are mostly surface sensitive. A handful of depth dependent methods exist in magnetism, among them neutron scattering and Nuclear Resonant Scattering (NRS), which is used in this study. NRS is a powerful experimental method^7^, possessing both sub nm depth resolution due to its isotope specificity and large penetration depth^8^ as well as the ability to resolve ordered magnetic structures on atomic length scales via diffraction methods^9^. In addition, NRS has been used to study magnetization dynamics in thin films^10^. Here, we use NRS to study dynamic spin structures in confined magnetic systems. Specifically, we study the nuclear resonant scattering process from a standing spin wave located in a microstructured permalloy stripe. The objective is to account for how the presence of a dynamic magnetization profile and the associated precession amplitude variation influences the obtained NRS signal. An incoherent superposition model in combination with an analytical description of the standing spin wave is able to explain the NRS data well, and allows us to reconstruct the dynamic spin configuration in the stripe. This sets the stage for future depth-dependent investigations of dynamic magnetization profiles in confined micromagnetic systems using NRS.

NRS involves a linearly polarized beam of 14.4 keV synchrotron radiation energy scattering from the energy levels of ^57^Fe nuclei. The degeneracy of the levels is lifted by the Zeeman interaction due to the hyperfine field present at the nucleus and all levels are excited simultaneously by the scattered photon. The responses of the excited nuclear resonances subsequently interfere with one another, causing a temporal beat pattern in the scattered intensity. Due to the long lifetime of the nuclear excited state (141 ns), the temporal response of the ^57^Fe nucleus can be readily detected. Under the assumption that the magnetic hyperfine field at the nucleus is collinear with the magnetization state of the sample, NRS can be used to probe the static magnetization state of a system^8^. A recent study has shown that the method is capable of mapping the spin precession trajectory in a permalloy film excited in the Kittel mode at GHz frequencies^10^. Here, we expand these studies to show that NRS is capable of reconstructing the dynamic magnetization profile of spinwaves in laterally confined microstructures. Important to this end is the fact that coherence properties of the beam influence the nuclear resonant scattering process from a system with lateral variation in the response to the synchrotron pulse. Specifically, we demonstrate the importance of the transverse coherence length (TCL) in interpreting nuclear resonant scattering data from laterally varying dynamic spin configurations. The relative size of the TCL with respect to the length scale of the variation in the spin configuration determines whether a coherent or incoherent superposition model is appropriate to describe the measured signal.

The microfocused beam used in this study has a TCL that is orders of magnitude smaller than the lateral length scale of variations of the dynamic spin structure of the standing spin wave. Consequently, the total NRS signal is the incoherent superposition of the coherent scattering signals from regions the size of the TCL across the dynamic magnetization profile. This total NRS signal carries information about the dynamic magnetization profile and is fit by an incoherent superposition model to reconstruct the spatial profile of the standing spin wave in the permalloy stripe. NRS can thus be used to probe laterally varying spin structures which opens new avenues in using NRS to study dynamic spin configurations in microstructured magnetic systems. Moreover, it highlights that control of the transverse coherence length is a prerequisite for accurate NRS data interpretation obtained from systems with laterally varying magnetizations.

## Results

**Figure 1:**
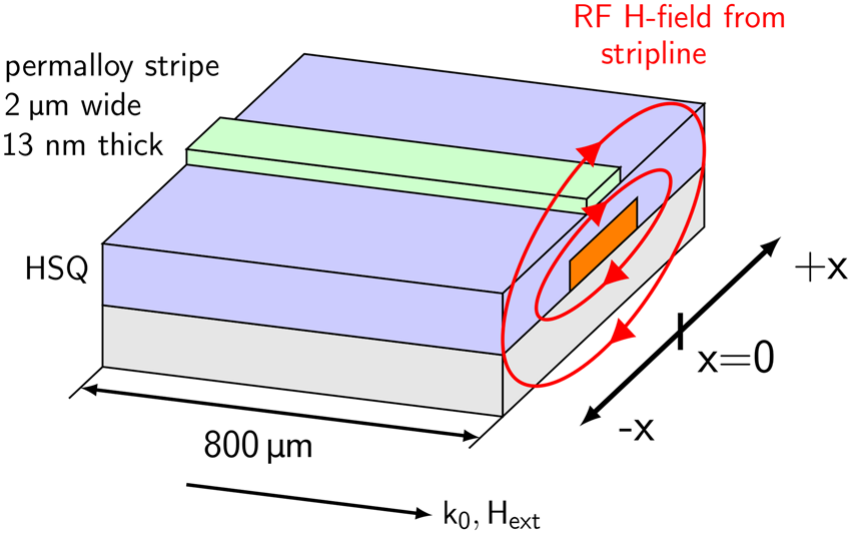
(**a**) Schematic representation of the sample layout. A dynamic magnetization profile with resonance frequency 2.65 GHz is induced in a 2 *μ*m wide Permalloy stripe by the radiofrequency field of a stripline. The external applied field *H*_**ext**_ and the incident wavevector *k*_0_ are oriented parallel to the stripe.

**Figure 2:**
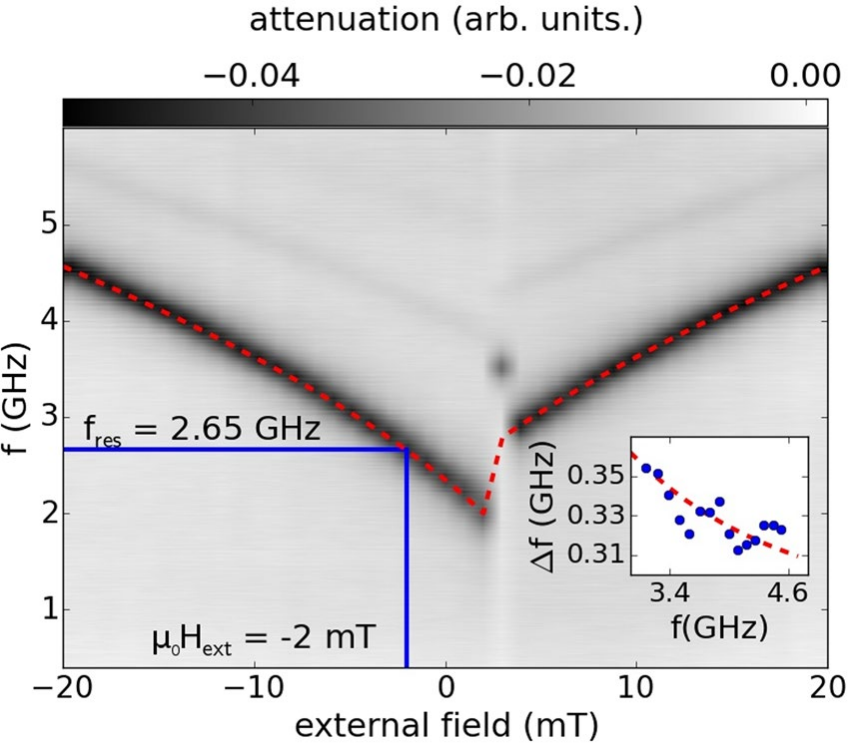
Absorption spectrum of the permalloy stripe as a function of external applied field and excitation frequency. The resonance frequency is set by the external field in the manner predicted by Eq. 1 (red dashed line). The inset shows the linewidth Δ*f* of the ferromagnetic resonance frequency as a function of frequency. The red dashed line shows that the Gilbert damping parameter of the magnetic system is *α* = 0.007. Higher order spin wave modes are visible in the absorption spectrum between 4.3 GHz and 5.4 GHz.

NRS data is obtained from a 2 *μ*m wide permalloy stripe excited by the magnetic GHz field of a gold stripline (see methods). A sketch of the sample setup is shown in Fig. 1. A vector network analyzer is used to measure the absorption spectrum of the sample as a function of the applied external field and excitation frequency (see Fig. 2)^11^. The magnitude of the high frequency magnetic field produced at the location of the permalloy stripe is $h=\frac{1}{2b}\sqrt{\frac{1}{50{\rm{\Omega }}}{10}^{\frac{{S}_{21}}{10}}P}$^12^ where *b* is the width of the stripe and under the assumption that the stripline is a sheet of current. *P* is the peak power input and *S*_21_ describes the absorption of the HF setup. The latter is approximately −2 dB at the ferromagnetic resonance frequency of the permalloy stripe.

High microwave absorption (black in the colorbar of Fig. 2) indicates a ferromagnetic resonance excitation in the permalloy stripe. As can be seen from Fig. 2, the resonance frequency is set by the magnitude of the static external field. The shape anisotropy of the stripe imparts a coercive field of 2 mT to the permalloy stripe that leads to an offset of the absorption spectrum along the vertical axis by approximately 1 GHz. The relationship between resonance frequency, the geometry of the stripe, and the external field is given by^13^

1 $${f}_{0}=\frac{\gamma {\mu }_{0}}{2\pi }\sqrt{({H}_{ext}+{H}_{A}+\frac{2t}{\pi w}{M}_{s})({H}_{ext}+{H}_{A}+(1-\frac{2t}{\pi w}){M}_{s})}$$

where *t* is the thickness and *w* the width of the permalloy stripe. The presence of perpendicular magnetic anisotropy due to surface roughness is neglected in this equation. A fit of the absorption spectrum using Eq. 1 is shown in Fig. 2. From the fit, we obtain a saturation magnetization of 740 kA/m and and the anisotropy field *H*_*A*_ = 2600 A/m. In the framework of the combined inhomogeneous broadening and Landau-Lifshitz Gilbert damping model^14^, the damping parameter is determined to be *α* = 0.007 (see inset of Fig. 2). A previous study^10^ reports a higher damping parameter due to spin pumping effects that are avoided in the present work.

**Figure 3:**
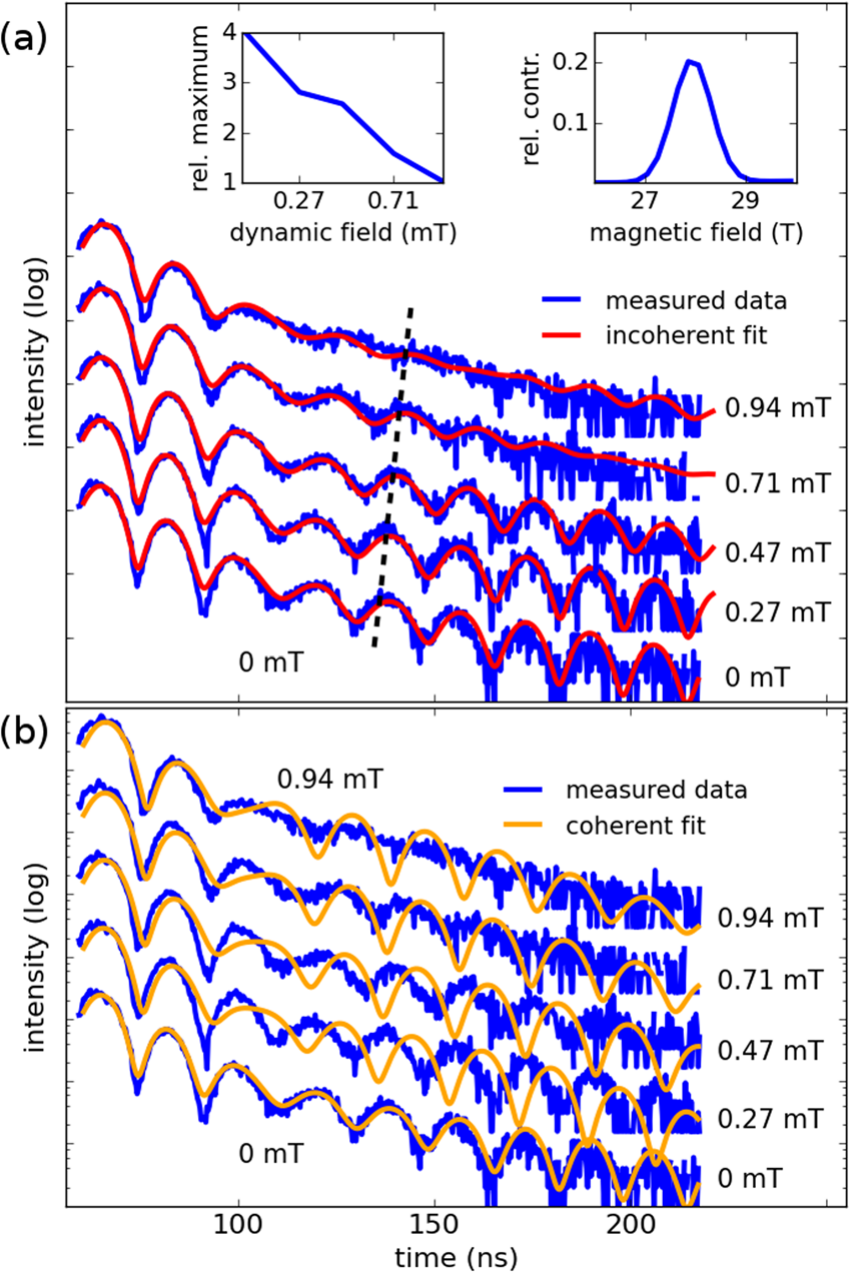
NRS time spectra taken from the standing spin wave excited at its resonance frequency of 2.65 GHz. The lowest time spectrum is taken without rf excitation. The dynamic field strength is noted on the right hand side of the spectra. As the excitation strength is increased, the time spectra are stretched and become blurred. The upper left inset shows a quantitative description of the blurring of the time spectra. The intensity of the 5th maximum (black dotted line) relative to the intensity of the 4th minimum is plotted in the inset. As the dynamic field strength is increased, minima and maxima at later times in the time spectrum become indistinguishable. The upper panel shows fits calculated by the incoherent superposition model explained in the text. The lower panel shows fits calculated by a coherent model for comparison. The right inset shows the deduced hyperfine field distribution in permalloy.

In this study, time spectra are taken from the permalloy stripe for increasing excitation field amplitudes at ferromagnetic resonance. The measured time spectra for increasing excitation field strengths are shown in Fig. 3. The time spectrum obtained from the permalloy stripe in the absence of an excitation field is also shown. This time spectrum allows determination of the hyperfine field distribution present in the permalloy. This distribution is shown in the inset of the upper panel of Fig. 3. The hyperfine distribution depends on the field at the positions of the Fe nuclei in the permalloy fcc crystal structure. Changes in the size of the crystallites vary the hyperfine field distribution experienced by the Fe nucleii. Variations in the size of the crystallites are induced by temperatures experienced by the sample during fabrication^15^. Slight discrepancies from literature values can thus be attributed to the sample fabrication process. As the excitation strength of the high frequency field exciting the permalloy stripe at ferromagnetic resonance is increased, the shape of the time spectra changes considerably. These changes in the time spectra with increasing excitation strength are twofold. First, a stretching of the extrema to later times is evident. As demonstrated^10^, this indicates a reduction in the hyperfine field distribution caused by the excitation of the stripe at ferromagnetic resonance. Second, an increased blurring of the extrema is observed as the excitation amplitude is increased. This blurring can be quantized by noting that the difference between adjacent minima and maxima in the time spectrum disappear as the dynamic field strength is increased. This trend is shown in the inset of Fig. 3. This trend of time spectra blurring as the dynamic field strength is increased is also evident in NRS time spectra obtained from 500 nm and 20 *μ*m wide permalloy stripes (see supplement). This blurring is not observed in time spectra obtained from a permalloy film excited in the Kittel mode, which does not exhibit a lateral profile in the magnetization dynamics^10^. In addition, a lack of blurring is observed in the time spectra obtained from a 240 *μ*m wide permalloy stripe, in which there are no strong spatial variations in the magnetization precession (see supplement).

## Discussion

**Figure 4:**
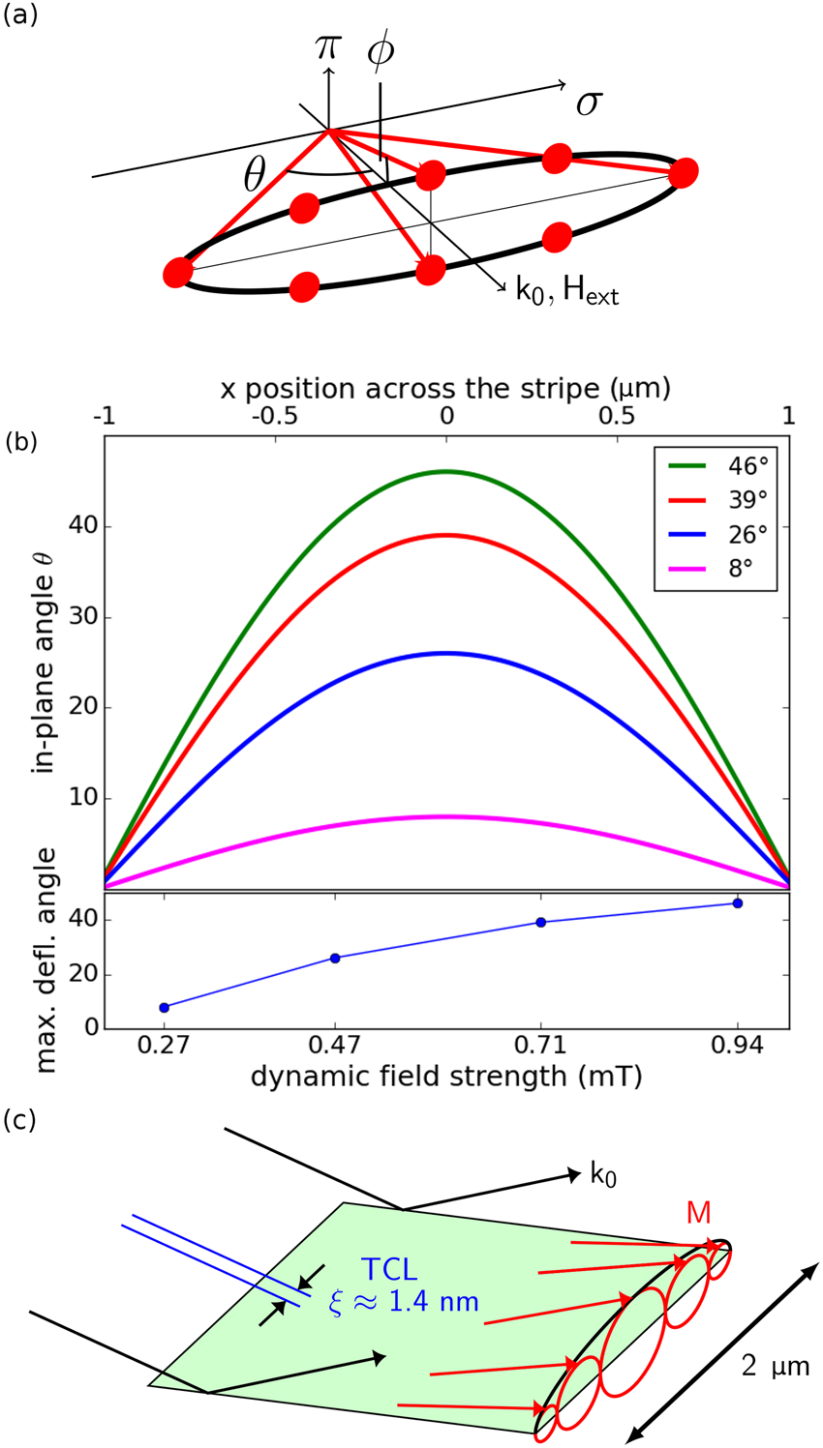
(**a**) Shows a schematic of the conical magnetization trajectory. The trajectory is parameterized by the in-plane opening angle (*θ*) and the out-of-plane opening angle (*ϕ*). The eight points along the magnetization trajectory used in the model are also shown as red dots along the conical trajectory. (**b**) Shows the in-plane deflection angle as a position(-dependent) function across the stripe as calculated by the analytical model of Guslienko^19^. Spatial profiles of the in-plane deflection angle for four maximum in-plane deflection angles are shown. The time spectra calculated on the basis of an incoherent superposition model based on these four spatial profiles best fit the obtained NRS data shown in Fig. 3. The lower panel of (b) shows the maximum deflection angle as a function of dynamic field strength as deduced from the fits in Fig. 3(a). The fit thus maps the spatial profile in the magnetization trajectory of the standing spin wave to a NRS time spectrum obtained at a specific dynamic field strength. (**c**) Shows a schematic representation of the magnetization dynamics profile of the standing spin wave located in the stripe. The amplitude of the magnetization precession depends on the position across the stripe. The TCL (*ξ* = 1.37 nm) of the beam is orders of magnitude smaller than the *μ*m length scale of lateral variation in the magnetization dynamics profile. As explained in the text, this requires an incoherent superposition model to describe the NRS data.

Previous studies mapping the Kittel mode in a permalloy film^10^ characterize the nuclear resonant scattering process using the stochastic model of Blume and Tjon^16^. This model describes the behavior of a quantum mechanical system whose Hamiltonian jumps between a finite number of possible states. In using this model to describe the magnetization precession on an elliptical trajectory, it is assumed that the magnetization orientation is collinear with the hyperfine field. In addition, it is assumed that the magnetization trajectory can be discretized into a finite number of points along the magnetization precession cone^10^. To model the spin precession present in both the Kittel mode in a film and the standing spin wave mode in a confined microstructure, eight magnetization directions are chosen as equidistant points along the elliptical precession path, each described by a different Hamiltonian for the magnetic hyperfine interaction (see Fig. 4(a)). Transitions are only allowed between neighboring points in such a way that precession direction is preserved. To determine the overall response of the system, the time scales relevant for the nuclear and magnonic systems must be compared in the framework of the coherent scattering model of Blume and Tjon.

The hyperfine transition energy of a nucleus in a uniform hyperfine field is^17^ ${E}_{{\bf{h}}{\bf{f}}}=({\mu }_{e}\frac{{m}_{e}}{{I}_{e}}-{\mu }_{g}\frac{{m}_{g}}{{I}_{g}}){B}_{{\bf{h}}{\bf{f}}}$, where *μ* is the nuclear dipole moment, *m* the magnetic quantum number, and *I* the spin state of the excited and ground levels respectively. For a Fe nucleus exposed to a hyperfine field of *B*_**hf**_ = 27.9 T (typical for a Fe nucleus in permalloy) this gives an intrinsic interaction time scale of the quantum system of approximately 100 ns. According to the model of Blume and Tjon, if the time scale of switching between the dynamic sites is shorter than the intrinsic period of the quantum system, it responds as if to the vectorial average of the sites that are used to describe the dynamic process^18^. As the precessional period of the magnetization trajectories of the first standing spin wave mode is approximately 350 ps, the response of the quantum system is to the vectorial average of the magnetization precession cone. Because the magnetization precession is point symmetric around the quantization axis, the vectorial average vector points parallel to the static external field. Although they are parallel, the effective hyperfine field vector during magnetic excitation has a smaller magnitude than the hyperfine field vector in the absence of excitation. This reduction in the magnitude of the hyperfine field vector causes a stretch in the nuclear quantum beats. The amount of reduction in the hyperfine field can be associated with a particular magnetization trajectory^10^.

This procedure has been used to determine the magnetization trajectory in a permalloy film excited in the Kittel mode^10^. Uniform precession of the film’s magnetization in the Kittel mode is associated with a stretch in the time spectra. Contrastingly, time spectra taken from the 2 *μ*m permalloy stripe excited at ferromagnetic resonance exhibit an increased blurring as the dominant effect when the excitation amplitude is increased (see Fig. 3). This is due to the dynamic magnetization profile that is induced in the excited permalloy stripe due to confinement. In a narrow, thin permalloy stripe it has been found that standing spin waves with cosine shaped lateral precession amplitude profiles form^19^. Such standing spin waves in confined permalloy microstripes have been well characterized both theoretically and experimentally^20^. Quantitative analysis of the effect caused by the dynamic magnetization profile on the time spectra can be performed in the context of describing the standing spin wave present in the permalloys stripe.

To develop a picture of the standing spin wave it is important to note that in the limit of a small aspect ratio of the stripe magneto static surface (Damon Eschbach) waves with wavevectors ${k}_{n}=\frac{(n+1)\pi }{w}[1-\frac{2}{d(p)}]$ form when the stripe is excited at resonance^19^. The effective pinning parameter *d*(*p*) depends on the mode number *n* and the aspect ratio *p* (*p* is thickness over width) of the stripe^19^ $d(p)=\frac{2\pi }{p[1+2\,\mathrm{ln}\,\frac{1}{p}]}$. The pinning of the magnetization at the stripe edges is due to the dipole interaction. Since the aspect ratio of the permalloy stripes investigated in this study is small, there is no phase variation in the spin precession along the stripe^21^. Rather, a spatial profile in the precession amplitude in the Kittel mode forms perpendicularly to the stripe (the profile develops in the x-direction in Fig. 1). The spatial amplitude variation has the form *A*(*x*) = *A*_0_ cos(*k*_*n*_*x*)^19^. The standing spin wave possesses higher order modes, which are visible in the absorption spectrum between 4.3 GHz and 5.4 GHz.

These analytic descriptions are used to produce a possible spatial profile of the standing spin wave present in the permalloy stripe studied here using NRS. The amplitude term *A*_0_ is treated as the maximum in-plane deflection angle (deflection angle in the x-direction in Fig. 1). The in-plane deflection angle increases as the dynamic field strength originating from the stripline is increased. The corresponding out-of-plane deflection angle is determined from the Smit-Beljers formulation^22^. The ratio of in-plane deflection angle *θ* to out-of-plane deflection angle *ϕ* is given by $\frac{\theta }{{\varphi }}=\sqrt{\frac{{H}_{ext}+{H}_{A}+(1-\frac{2t}{\pi w}){M}_{s}}{{H}_{ext}+{H}_{A}+\frac{2t}{\pi w}{M}_{s}}}=10.1$ for the system studied here. The precession cone parameterized by these angles is calculated for 400 equidistant points (5 nm spatial resolution) across the stripe. The precession cone profiles are calculated for maximum in-plane deflection angles from 1 degree to 50 degrees. Spatial profiles of the in-plane deflection angle for selected dynamic field strengths are shown in Fig. 4. The calculated precession cone profiles are instrumental in interpreting the NRS data shown in Fig. 3.

Using this calculated spatial profiles of the magnetization precession cones, the obtained NRS data are fitted with the coherent scattering model used in a previous work^10^. The fitting parameter is the maximum in-plane deflection angle of the precession cone spatial profile. The time spectrum calculated from a precession cone spatial profile is compared to the measured time spectra until a best fit is ascertained. Each time spectrum (for 0.27 mT, 0.47 mT, 0.71 mT, and 0.94 mT dynamic field strength) is compared to the time spectrum originating from a precession cone spatial profile (with maximum deflection angles from 0 to 50 degrees) until a best fit is achieved. However, the coherent scattering model is unable to account for the increased blurring due to the standing spin wave. A fit using the coherent model produced by the software CONUSS^23^ is shown in the lower panel of Fig. 3. In this model, a photon scatters coherently from an ensemble of permalloy unit cells each experiencing a hyperfine field corresponding to one of the magnetization trajectories at 400 equidistant points across the stripe. The trajectories are taken from the analytical calculations of the magnetization trajectories at points across the stripe. The intensity arriving at the detector is due to coherent scattering of the photon from the nuclear ensemble. The intensity is given by *I* = |*A*_1_ + *A*_2_ + *A*_3_ + … + *A*_*N*_|^2^ where *A*_*n*_ represents the nuclear decay associated with a particular magnetization trajectory. A key step to understanding why this coherent scattering model cannot accurately describe the scattering data is considering that the ^57^Fe nuclei in the stripe are exposed to different hyperfine fields and thus are distinguishable from one another. It has been shown that coherence properties of the beam influence the nuclear resonant scattering process from systems in which nuclei are laterally distinguishable^24^. If the transverse coherence length of the beam is smaller than the characteristic length scale of a transverse variation in the nuclear response, only an incoherent superposition model can correctly describe the NRS process. On the other hand, if the response does not vary laterally on a length scale much larger than the transverse coherence length only the use of a coherent scattering model is warranted. Thus an important aspect of an NRS experiment of a system with a laterally varying magnetization is knowledge and control of the transverse coherence length and use of the appropriate scattering model for correct data interpretation.

The transverse coherence length is given by^24^

2 $$\xi =\frac{\lambda }{2\pi }{(\sqrt{{(\frac{{\sigma }_{0}}{s})}^{2}+{(\frac{{\sigma }_{d}}{D})}^{2}})}^{-1}$$

where *σ*_0_ and *σ*_*d*_ are the size of the source and the detector, respectively. The distance between the source and the sample is given by *s*, and the distance between the sample and the detector is given by *D*. All scattering contributions coming from within a spatial region the size of the TCL add up coherently while contributions from outside of the TCL add up incoherently. For the setup used in this experiment the values were *σ*_0_ = 141.6 *μ*m, *s* = 41 m, *σ*_*d*_ = 1.5 cm, and *D* = 1.5 m. The calculated TCL for radiation at 14.4 keV is *ξ* = 1.37 nm, which is orders of magnitude smaller than the length scale of variations in the precession cone trajectory across the stripe. Consequently, the time spectra shown in Fig. 3 can be explained only with an incoherent superposition model. This model consists of two steps. First, the coherent scattering model previously described determines the time spectrum a certain magnetization cone would yield. In step two, step one is performed for each of the magnetization trajectories obtained by the analytical model for each of the 400 points across the stripe. Due to the standing spin wave, each point across the stripe is exposed to a different precession of the magnetization and thus yields a different nuclear response. The total nuclear resonant signal is then the sum of the temporal responses at the locations across the stripe. In contrast to a coherent scattering model, the intensity arriving at the detector is now the sum of the nuclear decays of nuclei exposed to different magnetization trajectories across the stripe *I* = |*A*_1_|^2^ + |*A*_2_|^2^ + … + |*A*_*N*_|^2^ where *A*_*n*_ is the nuclear decay associated with a region the size of the TCL. Across the stripe, these regions are exposed to different magnetization trajectories and thus yield different responses in the nuclear decay. The incoherent superposition of these responses correspond to the signal arriving at the detector.

To produce the incoherent fits in the upper panel of Fig. 3, the analytically calculated precession cone profiles are used in the incoherent superposition model previously described. Good agreement with experimental data is obtained using this approach. Especially the increased blurring of the time spectra with increased excitation strength is well described by the incoherent superposition model. Best fits are achieved for maximum in-plane deflection angles of 8, 26, 39, and 46 degrees for increasing dynamic field strengths (see Table in Fig. 4). The good quality of the fit suggests that the spatial contour of the standing spin wave in the stripe corresponds to the analytically calculated precession cone profile.

In this work we show that NRS can be used to reconstruct the spatial profile of dynamic spin configurations in microstructured magnetic systems. An important caveat is that this reconstruction requires additional spatial information provided by an analytical model. In the present work, knowledge of the mode number of the standing spin wave and the wave’s form provided by Guslienko’s analytical model described earlier are important prequisites to determining the exact spatial profile of the standing spin wave on the basis of NRS measurements. By being able to reconstruct spatial spin profiles in this manner, NRS is a complementary technique to a host of other methods common in magnetization dynamics including Brillouin Light Scattering, X-ray Magnetic Dichroism, and the Kerr Effect. The unique feature of NRS is its depth dependence due to the large penetration depth of X-rays at 14.4 keV and the method’s isotopic specificity. Further, the method has the distinct capacity to extract the three dimensional magnetization trajectory parameterized by in-plane and out-of-plane deflection angles^10^. In grazing incidence geometry the penetration depth is several 10 s of nm while in transmission geometry the penetration depth is up to several micrometers. The spatial lateral resolution of the method is given by the focus of the x-ray beam. At fourth generation synchrotrons the lateral resolution is expected to be at the diffraction limit. At Petra IV, for example, the diffraction limit is constrained by a minimum achievable wavelength of 1 *Å*^25^. This will enable a lateral resolution of down to one nanometer with hard X-rays. Of course, other synchrotron based methods such as XMCD would also benefit from the spatial resolution afforded by fourth generation synchrotrons. Temporal resolution of NRS is determined by how quickly changes in the time spectrum can be detected. Here, these changes are induced by the magnetization dynamics and result in a reduction in hyperfine field. Currently, the factor limiting temporal resolution is the gating electronics which separate the delayed resonant photons from the initial x-ray pulse. The best achievable temporal resolution is approximately 0.5 ns.

The measurement and their analysis presented here offer two important conclusions in the framework of expanding the method of NRS to probe magnetization dynamics. On the one hand, they make clear that key to studying inhomogeneous dynamic spin structures is knowledge of the TCL. As shown in this study, the relationship between the TCL and the length scale of spatial variations in the NRS response dictate whether a coherent or incoherent superposition model is to be used in data analysis. In magnetism and magnetization dynamics, numerous cases arise where samples exhibit laterally varying magnetic configurations which impart a spatial variation to the NRS response. Such configurations are present in magnetic domain patterns, micro- and nanostructed magnetic elements, and in systems with non-homogeneous spin dynamics. On the other hand, the study shows that NRS is capable of reconstructing dynamic spin configurations in microstructured systems. A prerequisite for this reconstruction is knowledge of the general spatial dependence of the magnetization trajectory provided by an analytical model or micromagnetic simulation. In combination with this additional information, NRS can be used to to give a detailed account of the spatial profile of the magnetization trajectory in confined systems. With the advent of fourth generation synchrotron sources, the drawback given by the beam’s large lateral focus (≈10 *μ*m) in combination with a small TCL (≈1.37 nm) which requires additional spatial information to unambigously identify a dynamic spin configuration will be eliminated. Synchrotron light at these sources will have an almost fully coherent beam with a nanometer focus even at hard X-ray energies. Consequently, NRS performed at these sources will be capable of lateral and depth dependent mapping of the magnetization dynamics with lateral nanometer and vertical sub-nanometer resolution.

## Methods

Nuclear resonant scattering experiments are performed in grazing incidence at the Dynamics Beamline P01 at PETRA III^26^ in Hamburg, Germany. The synchrotron is operated in 40 bunch mode with a bunch separation of 192 ns and a bunch duration of about 50 ps. The beamline P01 is equipped with a high resolution monochromator which reduces the energy bandwidth to approximately 1 meV around the 14.4 keV resonance of the ^57^Fe isotope. Kirkpatrick-Baez mirrors are used to focus the beam on the permalloy (Ni_80_Fe_20_) stripe that is 2 *μ*m wide and 13 nm thick. The beam is focused to 10 *μ*m in the horizontal and 5.7 *μ*m in the vertical direction. To excite the permalloy stripe, a 10 *μ*m wide gold stripline is fabricated on a GaAs substrate using electron beam lithography. The stripline is subsequently covered with 140 nm hydrogen silsesquioxane (HSQ) to provide a plane surface for the permalloy film and to ensure electrical insulation. The permalloy stripe, with a 13 nm thick Ta seed layer and 2 nm thick Ta capping layer, is then sputter deposited onto the HSQ and laterally defined by electron beam lithography. NRS measurements are performed just above the critical angle of total reflection of the Ta/Py/Ta system (≈0.25°) at which the beam footprint illuminates the entire permalloy stripe. NRS measurements are carried out in the Faraday geometry, in which the net magnetization of the permalloy stripe is parallel to the photon wavevector *k*_0_ as schematically shown in Fig. 1.

### Data availability

All data generated or analysed during this study are included in this published article.

## Electronic supplementary material

Supplementary Information
