# Supplementary material for: Incoherent Nuclear Resonant Scattering from a Standing Spin Wave
Source: Sci Rep. 2018 Jul 26;8:11261. doi: 10.1038/s41598-018-29596-z (PMC6062533; doi:10.1038/s41598-018-29596-z)
Supplement: Supplementary file 1 — Supplementary Information [file 41598_2018_29596_MOESM1_ESM.pdf]

Supplemental Material for Incoherent Nuclear Resonant Scattering from a Standing Spin Wave

J. Gollwitzer[1,*], L. Bocklage[2,3], K. Schlage[2], M. Herlitschke[2], H.-C. Wille[2], O. Leupold[2], C.F. Adolff[1,3], G. Meier[3,4], and R. Röhlsberger[2,3]

[1]Institute for Applied Physics, Universität Hamburg, Jungiusstrasse 11, 20355 Hamburg, Germany
[2]Deutsches Elektronen-Synchrotron DESY, Notkestrasse 85, 22607 Hamburg, Germany
[3]The Hamburg Centre for Ultrafast Imaging, Luruper Chaussee 149, 22761 Hamburg, Germany
[4]Max-Planck Institute for the Structure and Dynamics of Matter, Luruper Chaussee 149, 22761 Hamburg, Germany
[*]jakob.gollwitzer@desy.de

NRS time spectra from 500 nm, 20 μm, and 240 μm wide permalloy stripes were also obtained in this study. The stripes are excited by gold a stripline insulated by hydrogen silsesquioxane (HSQ). Time spectra obtained from the stripes are shown Fig. 1. Time spectra are taken when the stripes are excited at ferromagnetic resonance with a field strength of 1 mT (red curve) and in the absence of an rf excitation. In the time spectra obtained from the 20 μm and 500 nm stripes, blurring and stretching of the time spectra due to rf excitation is observed. This is indicative of the fact that a dynamic magnetization profile is present in the 500nm and 20 μm stripes.

Fig. 1(c) shows time spectra obtained from a 240 μm wide permalloy stripe excited by a 300 μm wide stripline. To obtain data from the 240 μm wide permalloy stripe, the beam is focused by Beryllium lenses to 300 μm in the horizontal and 120 μm in the vertical direction.The rf excitation induces a stretching in the time spectrum. The blurring induced in the time spectra of the 500 nm, 2 μm and 20 μm stripes by the rf excitation is not evident in the time spectrum of the 240 μm stripe. This allows one to conclude that a dynamic magnetization profile is not present in the 240 μm stripe and that it is excited in an essentially uniform Kittel mode. Indeed, micromagnetic simulations (see Fig. 2) confirm that for a 240 μm wide permalloy stripe, the magnetization trajectory does not vary significantly across the width of the stripe. Consequently, the data shown here support the conclusion that dynamic magnetization profiles can be resolved using NRS in conjuction with micromagnetic simulations as shown in the main body of the presented work.

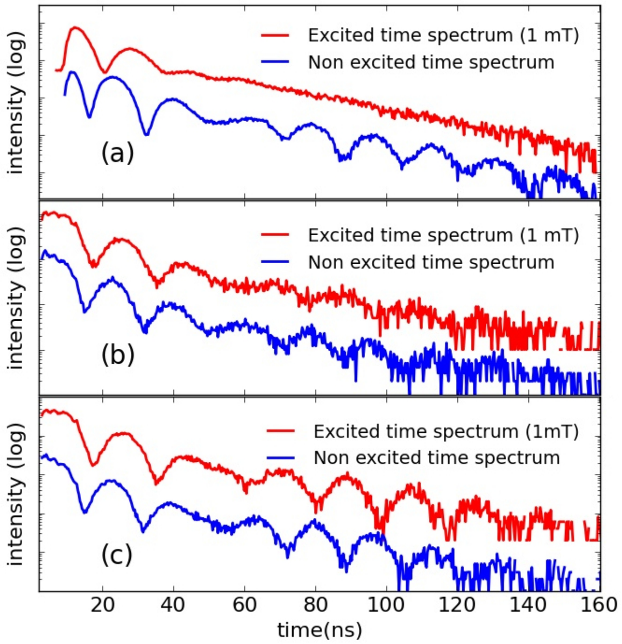

Figure 1. Shown are NRS time spectra obtained from (a) 500nm (b) 20 μm (c) 240 μm wide permalloy stripes. Time spectra taken at ferromagnetic resonance with an excitation field strength of 1 mT are shown in red. Note that the time spectrum from the 500 nm and 20 μm wide permalloy stripes exhibit blurring upon rf excitation while the time spectrum of the 240 μm wide stripe does not.

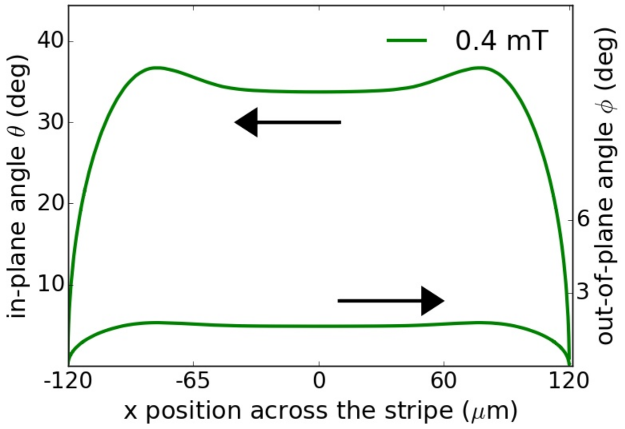

Figure 2. Micromagnetic simulation showing the in-plane and out-of-plane deflection angles as a function of position in a 240 μm wide permalloy stripe excited at ferromagnetic resonance by the radio frequency field of a gold stripline. Note that the deflection angles are essentially uniform across the stripe. This explains the lack of blurring in the time spectra obtained from the 240 μm wide permalloy stripe excited a ferromagnetic resonance.
